# FREDA: A Web Application for the Processing, Analysis, and Visualization of Fourier‐Transform Mass Spectrometry Data

**DOI:** 10.1002/rcm.9980

**Published:** 2024-12-30

**Authors:** David J. Degnan, Daniel M. Claborne, Amanda M. White, Sarah M. Akers, Natalie M. Winans, Yuri E. Corilo, Clayton W. Strauch, Vanessa L. Bailey, Lee Ann McCue, Kelly G. Stratton, Lisa M. Bramer

**Affiliations:** ^1^ Biological Sciences Division University of Chicago Illinois Chicago USA; ^2^ AI and Data Analytics Division Pacific Northwest National Laboratory Richland Washington USA; ^3^ Environmental Molecular Sciences Division Pacific Northwest National Laboratory Richland Washington USA

**Keywords:** exploratory data analysis, FT‐MS, statistics, web application

## Abstract

**Rationale:**

The high‐resolution measurement capability of Fourier‐transform mass spectrometry (FT‐MS) has made it a necessity for exploring the molecular composition of complex organic mixtures, like soil, plant, aquatic, and petroleum samples. This demand has driven a need for informatics tools to explore and analyze FT‐MS data in a robust and reproducible manner.

**Methods:**

FREDA is an interactive web application developed to enable spectrometrists to format, process, and explore their FT‐MS data without the need for statistical programming expertise. FREDA was built to explore outputs from a molecular identification tool, like CoreMS, and provide a suite of methods to filter data, compute chemical properties of peaks, statistically compare samples and groups of samples, conduct exploratory data analysis, and download the results with a report detailing all steps conducted.

**Results:**

To demonstrate the utility of FREDA, an example analysis was conducted using FT‐MS data from a soil microbiology study of samples collected in two different soil depths at the Sphagnum bog forest north of Grand Rapids, Minnesota. Differences between the two depths are observed using Kendrick, Gibbs free energy, and van Krevelen plots. G‐tests are used to quantify a significant difference between the groups. All analyses and plotting are conducted using only the FREDA application.

**Conclusions:**

FREDA is an open‐source and readily available web application that allows users to explore and make statistically valid conclusions about their FT‐MS data. The application is available online (https://map.emsl.pnnl.gov/app/freda) with a tutorial web series (https://youtu.be/k5HLE2kNSBY?si=yB6sGoyvzxrFf5MP) and freely accessible code on Github (https://github.com/EMSL‐Computing/FREDA).

## 1 Introduction

**FIGURE 1:**
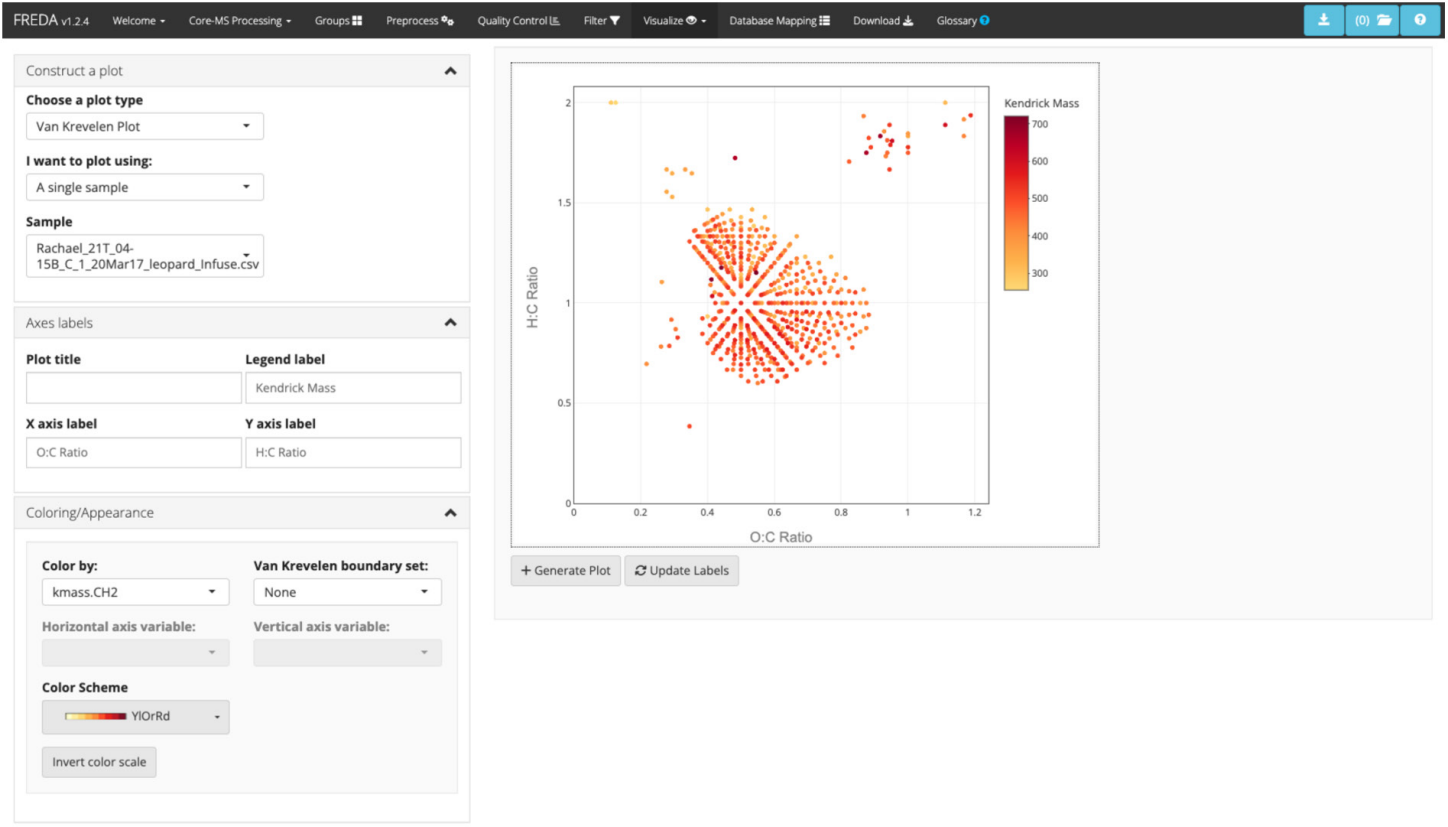
Example of a van Krevelen plot generated with FREDA on the *Visualize* tab. The top bar can be used to navigate a user through FT‐MS analysis. The side bar on the left allows users to set preferences for their plot, including axis labels and plot colors. The main portion of the app on the right shows an interactive plot where users can explore their data. Plots can be saved and revisited with the buttons on the top right of the application.

Fourier‐transform mass spectrometry (FT‐MS) platforms acquire high‐resolution and high‐mass accuracy measurements of the molecular composition of complex organic mixtures [1, 2, 3]. FT‐MS platforms have been used across a variety of complex mixture samples, including soils [4, 5, 6, 7], plants [8, 9], beverages [10, 11, 12], and aquatics [13, 14, 15]. The spectral data generated by FT‐MS instruments contain thousands to tens of thousands of observed peaks across a range of masses, and for high magnetic field instruments (e.g., 21 Tesla FT‐MS), it is not uncommon for hundreds of thousands of peaks to be measured, which necessitates the usage of bioinformatics tools for processing. In a typical FT‐MS workflow, following data acquisition from the instrument, an identification tool like CoreMS [16] is used to assign peaks to molecular formula. After identification, FT‐MS data are typically filtered, explored, and compared using downstream software tools, which can be data analysis coding packages [17, 18, 19] or web applications [20, 21]. An advantage of web applications is that they provide users the capability to process and visualize their own data without requiring users to learn programming. Thus, FREDA, the FT‐MS R exploratory data analysis tool, was built (Figure 1). Unlike existing web applications [20, 21], FREDA allows users to define groups of samples, use statistical tests to determine peaks unique to a group, customize their interactive plots, and map molecular formula to multiple databases (KEGG [22] and MetaCyc [23]). FREDA facilitates the formatting, processing, filtering, visualization, and statistical analysis of FT‐MS data in a series of steps. The application accepts output files from CoreMS [16] and also accepts a generalized format that supports data from any identification tool.

## 2 Software Methods

The underlying functionality for FREDA, the FT‐MS R exploratory data analysis tool, is contained in the ftmsRanalysis [19] R [24] package, available on github: http://github.com/EMSL‐Computing/ftmsRanalysis. FREDA is implemented as a web application using the R package shiny [25] and containerized with Docker [26] for hosting locally or on cloud servers. Instructions, links to tutorial videos, and downloadable example data are available through the web application on the *Welcome* tab and sub‐tabs.

In FREDA, the term “peak” refers to the *m/z* value of ions, which may or may not be assigned a molecular formula. These peaks may be either positively or negatively charged ions, which means that the specification of ion type is only relevant during steps conducted before FREDA, such as when a molecular formula annotation tool like CoreMS [16].

### 2.1 Data Upload

FREDA allows users to upload data in one of two formats: (1) the output files from FT‐MS identification in CoreMS [16], where each file is a sample; or (2) a generalized input consisting of two files—the “data file” and the “molecular identification file.” If uploading data from CoreMS, users are taken to the *Create CoreMS* sub‐tab in the *CoreMS Processing* tab where users will first review the auto‐detection of column names and revise if needed. In the *Confidence Filter* sub‐tab, users can determine an appropriate confidence score filter for their data, where the objective is to minimize mass errors while maximizing the number of peaks retained. The mass error plot and table summary on this page can be used to determine a reasonable cut‐off. Finally, in the *Formula Assignment* sub‐tab, users can pick an appropriate molecular formula based on the highest confidence score or peak height from the list of molecular formula calculated by CoreMS.

If using the general input format, the “data file” contains the peak intensities for each peak in a sample, where rows correspond to the masses and columns correspond to the samples. One column should contain the mass identifiers, and all other columns should contain the abundances. Note that if users would like to compare the same analytical replicates of a sample, they can add each replicate as a column in the “data file.” The “molecular identification file” should contain the same masses as in the “data file,” with additional information, including the molecular formulas or the C, H, O, N, S, and P counts. C and H are required. Isotope forms (e.g., C13) are also supported.

For both input formats, users then specify the scale of their data, what values denote missingness (typically an abundance of 0), and whether they would like to filter isotopes. After a successful data upload, summaries of the data, such as the number of peaks per sample, are provided.

### 2.2 Specifying Groups

The *Groups* tab allows users to assign samples to one or more experimental groups of interest. For ease of group assignment, group names can be detected from sample names with an easy‐to‐use text matching module where users specify a pattern to detect in column names. Downstream visualization and analyses use these groups whenever appropriate. For example, two groups can be compared to determine peaks uniquely observed in each group and plotted in the *Visualize* tab.

### 2.3 Preprocessing

The *Preprocessing* tab provides options to compute several chemical properties, including elemental ratios (e.g., O:C), Kendrick mass and defect [27], nominal oxidation state of carbon (NOSC) [28], Gibbs free energy (GFE) of the carbon oxidation half reaction under standard conditions (GFE) [29], aromaticity index [30], double‐bond equivalent (DBE) [30], counts of elemental composition [4, 31, 32], and compound classes [4, 31, 32] (e.g., amino sugars, carbohydrates, etc.), whenever applicable. By default, the ratios of elemental counts and Kendrick mass and defect are selected, as these are required to produce van Krevelen and Kendrick plots in the *Visualize* tab. These properties and the equations used to calculate the values are described in FREDA's glossary, which is the last tab in the tab bar (Figure 1). After values are calculated, summary tables of these quantities are displayed, and a graphical summary of the calculated values across all samples can be generated via a dropdown selection menu.

### 2.4 Quality Control

The *Quality Control* tab provides a visual overview of peak distributions with boxplots and a bar chart showing the number of observed peaks. Users can graph the data by sample and display the raw intensity, log transformed intensity (log2, log10, and natural log), or presence/absence values. By default, all samples are shown, but graphs can be filtered by group if groups were defined in the *Groups* tab.

### 2.5 Filtering

Four predefined but optional filters are available, along with the ability to add up to three custom filters. If using the predefined filters, users may filter by sample name, a mass range, a minimum number of non‐missing observations (per group, if specified), or by peaks with an associated molecular formula. Custom filters can be used to filter peaks by any value from the “molecular identification file” or values that were calculated in the preprocessing tab. The filters can easily be reset with the “Reset Filters” button.

### 2.6 Visualization

The *Visualize* tab supports the creation, display, and saving of plots. Saved plots can be downloaded in the *Download* tab. The left side panel of the *Visualize* tab contains dropdowns where the user can specify plot type [van Krevelen, Kendrick, Density, principal coordinate analysis (PCoA), or a custom scatter plot], plot content (a single sample, multiple samples, a comparison of groups, or a comparison of two samples), and plot modifications such as editing the plot title, legends, and axes, specifying colors according to data values and configuring the color scale.

#### 2.6.1 Comparing Groups

It is often of interest to determine whether a peak is unique to one of the experimental groups, and FREDA provides two options for this determination. One method (presence/absence thresholds) uses thresholds corresponding to the proportion of observations in each group to determine whether a peak is considered “observed” or “absent.” The second method uses a G‐test [33] to test the hypothesis that the probability of observing a peak is independent of the group members. The G‐test is analogous to a chi‐square test of independence with a correction for small group sizes and requires that groups consist of at least three samples each.

#### 2.6.2 Plot Types

All plots in FREDA are interactive plotly [34] graphics, which allow users to zoom in and out, select groups of data points to display or hide, and view additional information about data points by hovering over them. Five primary plot types are available in FREDA and explained here in further detail. First, van Krevelen plots display each peak based on the O:C (*x*‐axis) and H:C (*y*‐axis) ratios. Users may color these plots by any other values, such as N:C ratio if the user calculated this value in the *Pre‐Processing* tab. FREDA can highlight peaks unique to a particular group or those observed in both, as determined by either the presence/absence thresholds or the G‐test. Second, Kendrick plots display each peak based on Kendrick mass and defect on the *x*‐ and *y*‐axis, respectively. Hovering over a specific point in the van Krevelen and Kendrick plots will give its molecular formula and mass. Third, density plots show the distribution of a specified variable across observed peaks in the selected sample. Fourth, PCoA plots display samples by any two of the first five principal components from a PCoA decomposition. Users may choose the distance metric for PCoA to find where the percentage of variance explained on the first two principal components is highest. These values are reported as an *R* ^2^. Finally, users may specify a custom scatter plot with any “molecular identification data” or preprocessing variables on the *x*‐ and *y*‐axis.

#### 2.6.3 Saving and Linking Plots

Buttons in the top right of the application can be used to save each individual plot, and the saved plots can be compared using the *Linked Plots* sub‐tab under the Visualize tab. First, select two saved plots to compare in the table under “Choose Two Plots to Compare.” A selected row will highlight blue. Click the “Compare These Plots” button to interactively visualize the plots. Selecting a region in one plot will highlight the corresponding data in the other plot for ease of comparison.

### 2.7 Database Mapping

Molecular formulae can be mapped to their reactions, modules, and pathways from both KEGG [22] and MetaCyc [23]. To limit the number of entries returned, specify a maximum number of records returned per molecular formula with a default maximum of five. Data can be returned in wide format, where each entry is separated by semicolons in a long text string, or in long format, where each entry is returned in its own row. This specification can be made with the “Make unique rows for which variable?” picker widget. Generated tables can be saved and downloaded in the *Download* tab.

### 2.8 Download

Following visualization, users can download their files and saved figures as a compressed archive. An HTML summary report is generated automatically and contains information about the original uploaded data, the selections that were made on the upload tab, the calculated values from preprocessing (with equations), and the filters applied to the data. Modified versions of the two original uploaded files that reflect all changes made in FREDA can be downloaded in CSV format, either separately or in a merged sheet. If analyses were performed on more than a single sample, then there is an optional download for CSV files summarizing the data in a group and the results of uniqueness tests performed in the *Visualization* tab. At any point in the FREDA application, saved plots can be viewed and removed with an optional menu that pops up when clicking the folder icon in the top right of the application.

## 3 Results and Discussion

The capabilities of FREDA were demonstrated using data from a soil experiment conducted at the Sphagnum bog forest north of Grand Rapids, Minnesota [35]. A total of 50 samples and 17 soil cores were taken, representing samples originating from three sample depths (0.1, 0.4, and 0.75 m) and two extraction strategies: chloroform and methanol. Here, peaks were compared at each depth for the chloroform extraction.

**FIGURE 2:**
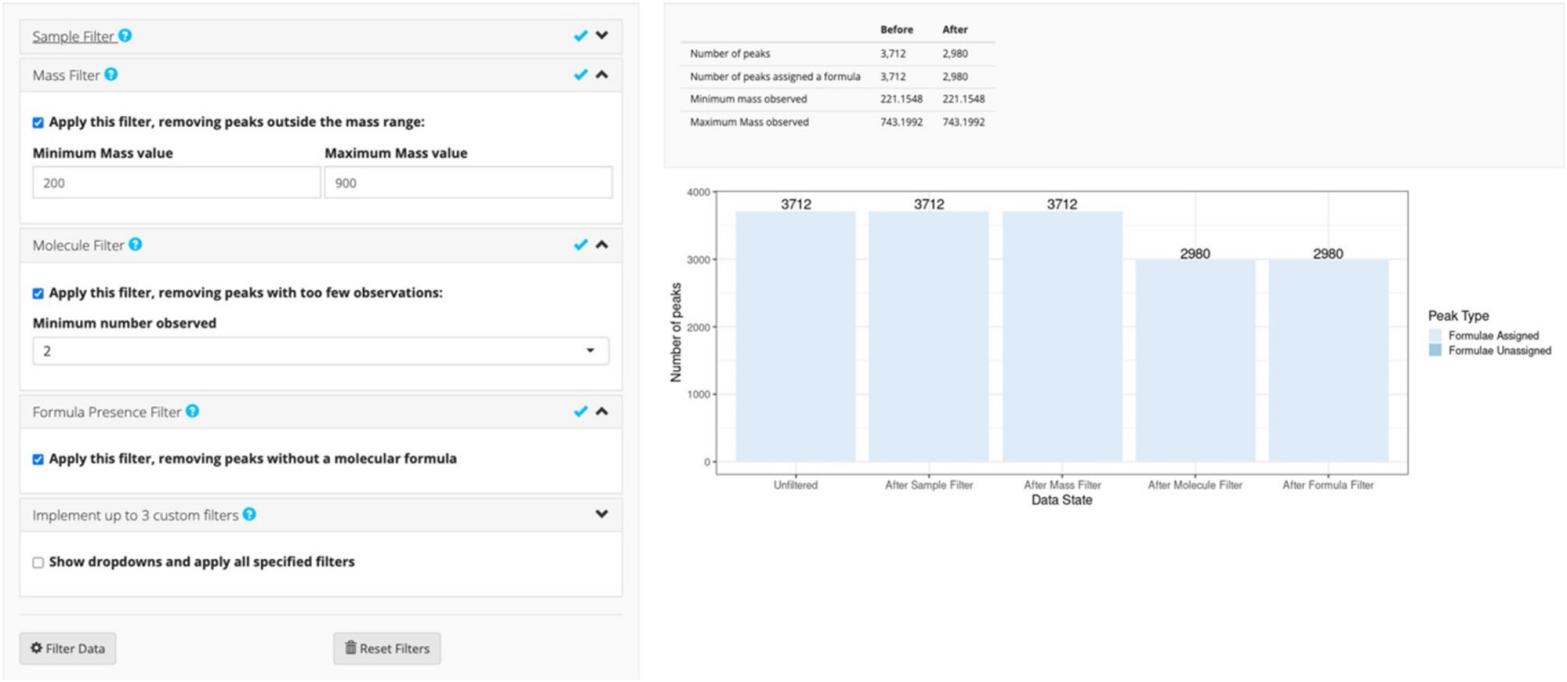
The *Filter* tab in FREDA. Filters and their filter ranges are selected on the left panel. Information on peaks removed and formula assignment are displayed in the table on the top right. Bar charts show the number of peaks with formula assignment (light blue), and the number of remaining peaks after each filter is applied.

Characterization of the soil organic matter (SOM) in these samples was carried out using an Agilent 21 Tesla Fourier‐transform ion cyclotron resonance (FTICR) mass spectrometer at the Environmental Molecular Science Laboratory (EMSL) in Richland, Washington. Molecular formulas for peaks were obtained using the identification tool CoreMS [16]. The data were uploaded to FREDA and samples were assigned to their respective groups in the *Groups* tab. A total of 3712 peaks were observed across the 50 samples. Three filters were applied: a mass filter removing peaks with a mass outside of the range [200, 900], a molecule filter removing peaks that were not observed in at least two samples, and a formula filter removing peaks with no assigned formula. Figure 2 shows the *Filter* tab for these data, where the bar chart shows the effect of each filter applied sequentially to the data. The application of the filters resulted in a final dataset consisting of 2980 peaks.

**FIGURE 3:**
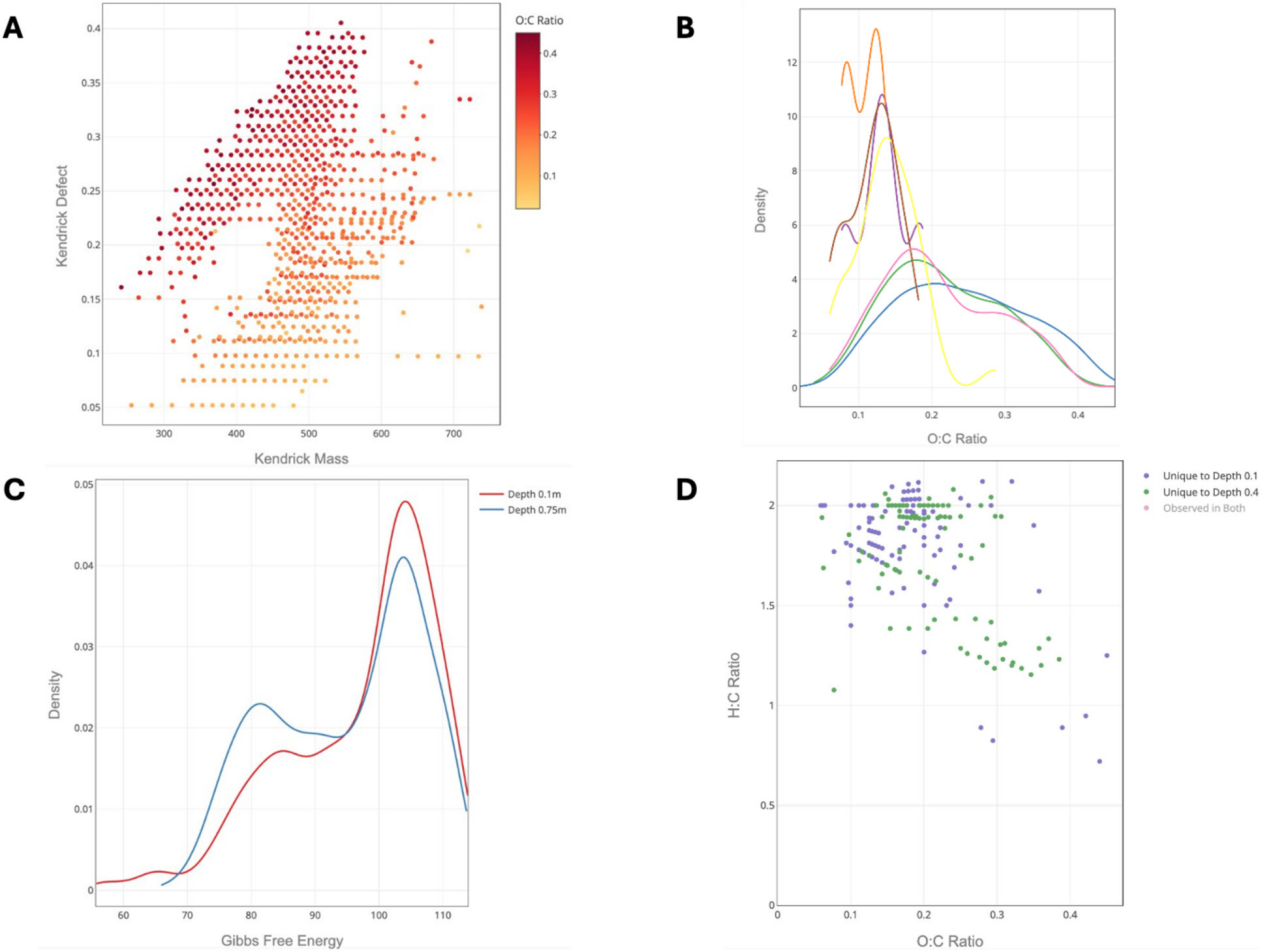
Selected plots from the chloroform extraction available in the *Visualize* tab of FREDA. (a) A Kendrick plot of a single sample with peaks colored by the O:C ratio. (b) Density curves of the observed O:C ratios for each sample in the 0.75‐m depth group. (c) Density curves of the GFE for the depth at 0.1‐m and depth at 0.75‐m groups. (d) van Krevelen plot of the uniquely observed peaks for the depth at 0.1‐m and depth at 0.4‐m groups, as determined by the G‐test.

The *Visualize* tab was then used to plot samples in the chloroform extraction group, including a Kendrick plot with peaks colored by their respective O:C ratio (Figure 3a). The molecular formula can be viewed by hovering over a plotted peak. Within‐group properties were investigated via all plot types except the custom scatterplot. Figure 3b shows the density of observed peaks' O:C ratios for each sample from the depth at 0.7‐m group. The combined density of all samples in the group can be viewed, but is toggled off in the interactive plot shown. Four of the seven samples have O:C ratio densities shifted toward lower ratios compared to the other three samples. Density plots were also used to examine the calculated properties of the observed peaks. Figure 3c shows the distribution of GFE values, an indication of energetic potential, observed across samples in the depth at 0.1‐m and depth at 0.75‐m groups. The samples at 0.1 m have higher GFE values compared to the samples at 0.75 m.

To determine which peaks are uniquely observed when comparing the depth at 0.1‐m and depth at 0.4‐m samples, FREDA was used to perform a formal statistical test, the G‐test. Figure 3d shows a van Krevelen plot of the peaks unique to both groups, omitting peaks observed in both groups (“observed in both” in the figure legend has been toggled off). This visualization shows differences in H:C and O:C ratios between the two groups.

Many other exploratory plots and analyses are available in FREDA in addition to those highlighted above. The *Download* tab shows a table including entries for the plots created throughout the FREDA workflow, as well as entries for the processed data and metadata and the data underlying our group comparison plots. This information includes the results of the group comparison by G‐test, specifying the group, if any, in which a peak was uniquely observed. Users can select which of these items they would like to download, and report files that track all the calculated values, filters, and plots of interest.

## 4 Conclusion

FREDA is an open‐source web application for processing and comparing FT‐MS data in a reproducible and citable workflow. Though FREDA is streamlined to directly support output files from the identification tool CoreMS [16], the generalized format allows for easy integration with other FT‐MS formula identification software. For those interested in implementing FREDA's functionality for code‐based pipelines, all functionality currently exists within the R package ftmsRanalysis [19]. The open‐source implementation of FREDA allows for the addition of new capabilities, analysis methods, and plot types as needs arise.

## Author Contributions

**David J. Degnan:** writing – original draft, writing – review and editing. **Daniel M. Claborne:** visualization, software, writing – review and editing. **Amanda M. White:** software, visualization, writing – review and editing. **Sarah M. Akers:** visualization, writing – review and editing, software. **Natalie M. Winans:** software, writing – review and editing. **Yuri E. Corilo:** writing – review and editing. **Clayton W. Strauch:** software, writing – review and editing. **Vanessa L. Bailey:** writing – review and editing, data curation. **Lee Ann McCue:** data curation, conceptualization, writing – review and editing. **Kelly G. Stratton:** writing – review and editing, supervision. **Lisa M. Bramer:** conceptualization, funding acquisition, writing – review and editing, software, formal analysis, project administration, supervision.

## Data Availability

The data that support the findings of this study are available in National Microbiome Data Collaborative at https://data.microbiomedata.org. These data were derived from the following resources available in the public domain: ‐ National Microbiome Data Collaborative, https://data.microbiomedata.org/details/study/nmdc:sty‐11‐33fbta56.
